# The Relationship Between Seasonal Variation in the Diagnosis of Acute Lymphoblastic Leukemia and its Prognosis in Children

**DOI:** 10.5505/tjh.2012.12244

**Published:** 2012-06-15

**Authors:** Mehmet Mutlu, Erol Erduran

**Affiliations:** 1 Karadeniz Technical University, School of Medicine, Department of Neonatology, Trabzon, Turkey; 2 Karadeniz Technical University, School of Medicine, Department of Pediatric Hematology, Trabzon, Turkey

## TO THE EDITOR

Although many studies have examined the prognostic factors of childhood ALL, a limited number have investigated the correlation between the season and/or month in which ALL is diagnosed and its prognosis [1,2]. Herein we report on the correlation between the month and season in which ALL is diagnosed and its prognosis in children. This retrospective study included pediatric ALL patients that were treated at the Karadeniz Technical University, Pediatric Hematology Department between 1990 and 2004. Cases were reviewed in terms of the month and season ALL was diagnosed, and prognosis. Patients were treated with the St. Jude Total XI or XIII treatment protocol. 

The study included 137 patients; 81 were male (59%) and 56 (41%) were female. Mean age of the patients was 5.27 ± 3.86 years (range: 8 months-16 years) and 84% (n = 116) were aged 1-10 years. Most of the ALL patients were diagnosed in March (16%, n = 22), followed by July (13.9%, n = 19) and September (13.9%, n = 19) (Table 1). There weren’t any statistically significant differences between the months and seasons in which ALL was diagnosed (P > 0.05). Patients diagnosed in September had the lowest mortality rate (21%), followed by those diagnosed in November (27%) and October (37%); patients diagnosed in December and January had the highest mortality rate (80%) (Table 1). Most of the patients (31%) were diagnosed with ALL during spring, whereas only 15% were diagnosed during winter. The highest cure rate was observed in the patients diagnosed during autumn (74%) and the highest mortality rate (67%) was among those diagnosed during winter (P = 0.023) (Table 2). In all, 56% (n = 77) of the patients were cured and 44% (n = 60) died. 

Seasonal distribution of the diagnosis of ALL according to month and season has been extensively investigated, though results are inconsistent [2-8]. Ross et al. [5] reported that there was a notably higher incidence in some months (May [8.9%], July [9.0%], and August [9.0%]) and that a statistically significant peak was observed during summer (26%) in the northern US. Westerbeek et al. [7] reported peak incidences in May [10%] and August [9.3%]. Researchers have speculated that these results may constitute corroborative evidence of an infectious etiology for ALL. Karimi and Yarmohammadi [4] reported that the diagnosis of ALL peaked in November (P < 0.05) and during autumn (29%, n=62) (P > 0.05); they suggested that there is seasonal variation in the diagnosis of ALL and that infections may play a role in the progression of ALL, rather than in its onset. Gao et al. [3] reported that there wasn’t seasonal variation in the diagnosis of ALL in Singapore or the US, although a significant peak was observed in early January in western Sweden. They also reported that there was a lack of strong evidence for the role of climate in the incidence of ALL. Cohen [1] reported that there weren’t any statistically significant differences between seasons of onset, whereas Haris et al. [9] reported trimodal patterns in the US, with peaks in April, August, and December at locations above 40° latitude, and in February, July, and October below 40° latitude. 

The present study was conducted in a region located between 39°45¢ and 41°45¢ north. The pediatric ALL cases were more often diagnosed in March [16%], July and September [13.9%] and seasonally during spring [31%], but there were no statistically difference between the months and seasons. The present findings are in agreement with those of Haris et al. [9]. Haris et al. [9] reported that seasonal factors (environmental allergens and/or infectious agents) promote leukomogenesis via indirect mechanisms. We think that these peaks may coincide with seasonal elevation of allergic factors during spring and infections caused by such viruses as influenza during winter. But we have no idea about cases diagnosed in summer. Meltzer and Annegers [2] suggested that there wasn’t a correlation between the season in which treatment was initiated and survival rates. As such, the timing of the commencement of the treatment of ALL is not a prognostic factor. On the other hand, Cohen [1] reported that the survival rate among patients diagnosed between November and January was 56.5%, as compared to 80% among those diagnosed between August and October. In the present study the survival rate among the patients diagnosed during winter was 33% and the most favorable prognosis was in patients diagnosed during autumn (74%). Viral infections are common during winter and may be associated with an increase in relapses in ALL patients. 

In conclusion, we think that ALL might vary seasonally in diagnosis and prognosis, but the our results were not statistically significant because of the small study group, which is a limitation of the study. We think that the time at which ALL is diagnosed may be a valid prognostic criterion in pediatric ALL patients. 

**Conflict of Interest Statement **

The authors of this paper have no conflicts of interest, including specific financial interests, relationships, and/ or affiliations relevant to the subject matter or materials included.

## Figures and Tables

**Table 1:**
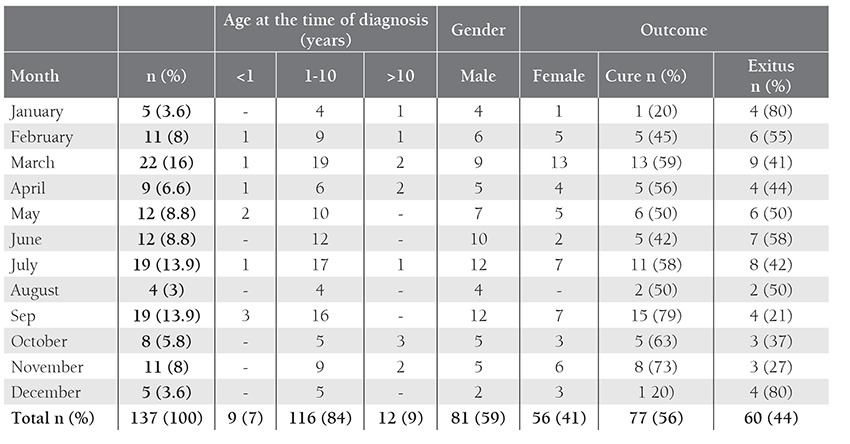
Distribution of the patients according to the month ALL was diagnosed and prognosis.

**Table 2:**
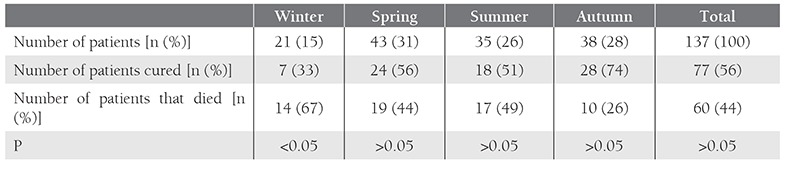
Seasonal distribution among the pediatric ALL patients and their outcomes.
